# Distribution and molecular characterization of integron classes from *Escherichia coli* and *Klebsiella pneumoniae* isolates in Sulaymaniyah province of Iraq

**DOI:** 10.1128/spectrum.01751-25

**Published:** 2025-09-10

**Authors:** Taib Ahmed Hama Soor

**Affiliations:** 1 Medical Laboratory Department, College of Health and Medical Technology, Sulaimani Polytechnic University 467127, Sulaymaniyah, Iraq; 2 Department of Medical Laboratory Analysis, College of Health Sciences, Cihan University - Sulaimaniya 584270, Sulaymaniyah, Iraq; Rowan University Cooper Medical School, Camden, New Jersey, USA

**Keywords:** integron, antibiotic resistance genes, *Enterobacteriaceae*, environmental pollution

## Abstract

The environmental pollution from the misuse of antimicrobial drugs is fueling selection pressure in bacteria, thereby exacerbating the threat to global health. In Iraq, the situation is made worse by the poor implementation of the World Health Organization’s Global Antimicrobial Resistance and Use Surveillance System (WHO-GLASS). Consequently, this study aimed to increase surveillance of the spread of antimicrobial resistance in Sulaymaniyah, Iraq. A total of 296 *Enterobacteriaceae* comprising 147 *Klebsiella pneumoniae* and 149 *Escherichia coli* were isolated from humans, poultry, and dairy farms. The isolates were screened using multiplex PCR to assess the prevalence of the clinically important integron integrase (*intI*) classes and antimicrobial resistance genes (ARGs) of commonly used antibiotics. Remarkably, 81.14% of the isolates carried at least 2 ARGs, 10.47% *intI1*, and 3.72% *intI2*. No *intI3* was detected. A total of 663 ARGs were identified using multiplex PCR in the two *Enterobacteriaceae*: beta-lactamase genes were 43%, tetracycline resistance genes 25.20%, sulfonamide resistance gene 16.10%, quinolone resistance gene 10.2%, and aminoglycoside resistance genes 5.7%. *K. pneumoniae* harbored more integrons and ARGs than *E. coli*, thus posing a higher antimicrobial resistance threat in this province. This study underscores the importance of implementing more stringent WHO-GLASS and antibiotic stewardship to end the multidrug resistance crisis in Iraq.

**IMPORTANCE:**

These data are about the prevalence of integrons and resistance genes, helping to fill a significant gap in global surveillance efforts. Results can be used by global health authorities and the World Health Organization to develop national and international antimicrobial resistance (AMR) control strategies. The study is important because integrons are key genetic platforms that capture and disseminate antibiotic resistance genes among bacteria. In addition, *Escherichia coli* and *Klebsiella* spp. are among the top causes of hospital- and community-acquired infections, especially urinary tract infections, bloodstream infections, and pneumonia. Therefore, it will be riskier when these bacteria have a high rate of integrons and resistance genes because it impedes treatments during infection. Another importance of this study is that the study was carried out in Iraq. Iraq, like many low- and middle-income countries, faces challenges with unregulated antibiotic use, leading to high rates of AMR.

## INTRODUCTION

The widespread, unrestricted use of antibiotics in agricultural practices, animal husbandry, and the prevention of infections in humans has enabled the development of multidrug resistance in bacteria (1). Thus, understanding the challenges of antimicrobial resistance development and spread requires a holistic investigation of these bacterial reservoirs in the environment (2). While antimicrobial resistance is a natural phenomenon, the current surge in multidrug resistance is due to selection pressure exacerbated by the unrestricted usage of antibiotics (3, 4). Bacteria under antibiotic selective pressure will undergo random mutagenesis in their chromosomes to produce progenies that could survive or acquire resistance genes from within the environment through mobile genetic elements (5).

The open reading frames of the acquired antimicrobial resistance genes (ARGs) are translated into proteins with various modes of action. The likes of chloramphenicol acetyltransferases and beta-lactamases eliminate or directly change the antibiotics. In some, the antibiotic’s target site could be altered to prevent effective binding of the drug. The open reading frames translation of some could diminish the permeability of the cell membrane to the drugs. Others are translated as efflux pumps that aggressively pump the drug out of the cell (6). These ARGs are disseminated among bacteria within the environment by mobile genetic elements (7). The DNA elements, such as plasmids, transposons, and integrons, are therefore important drivers of the spread of ARGs.

Integrons have been implicated as the main mobile DNA elements responsible for the horizontal transfer of AMR genes between bacteria within the environmental microbiota (8). Integrons’ DNA structural composition consists of site-specific recombination system of encoded integrase and *attI* recombination site and integron integron-associated promoter; thus, these features allow the acquisition, expression, and dissemination of gene cassettes: an array of multiple AMR genes coupled with the site-specific *attC* DNA sequence. In general, three clinically important integron classes have been identified based on the DNA sequences of their associated integrases: class 1, class 2, and class 3 (9). Surprisingly, two classes of these integrons are commonly found among gram-negative bacteria such as *Escherichia coli* and *Klebsiella pneumoniae*, which are responsible for conferring resistance to different classes of antibiotics (10). Class 1 integron is the most abundant in clinical bacteria (11). It has a conserved 5′ region consisting of the integron-integrase 1 with promoters and the binding site for recombination, and the 3′ conserved region with DNA sequences of *qacEΔ1* and *sul1 genes*. In between these two conserved regions is the variable region where captured genes for expression are processed (11)

*Enterobacteriaceae* are a group of gram-negative bacteria that are found in the intestinal tract of humans and animals as opportunistic pathogens. Members of this family frequently cause infections and hospital-acquired infections, such as bloodstream infection, pneumonia, and abdominal infection. In clinics, this family attracts the attention of scientists and clinicians globally because of their ability to gain multiple drug resistance (12–14). *E. coli* and *K. pneumoniae* are responsible for many epidemiological and nosocomial infections (15). Both *K. pneumoniae* and *E. coli* have been reported to harbor extended-spectrum beta-lactamases (ESBLs), resistance genes responsible for inactivating beta-lactam antibiotics (16, 17).

In Iraq, the surveillance system is inadequate for the distribution and monitoring of antibiotic usage; thus, misuse and indiscriminate consumption of antibiotics are inevitable, with the consequential selection pressure on the disease-causing bacteria to survive in the drug-polluted environment (18, 19). Subsequently, evaluating the prevalence of ARGs and the classes of integrons encoded in *K. pneumoniae* and *E. coli* from clinical samples of humans, animals, and the environment is critical to ending antimicrobial resistance in Iraq. Hence, this study was carried out to evaluate the occurrence of antimicrobial genes and integrons in *K. pneumoniae* and *E. coli* isolates from clinical samples of animal, poultry, and human as well as in healthy hosts in the city of Sulaymaniyah, Iraq.

## MATERIALS AND METHODS

### Sample collection and bacterial isolation and identification

A total of 296 gram-negative bacterial isolates from 149 *E. coli* and 147 *K*. *pneumoniae* were collected and evaluated in this study in 2024. The *Enterobacteriaceae* were isolated from humans and livestock, such as chicken, pajeon, sheep, goat, cattle, and rabbits suffering from various infections. The samples were processed in the laboratories of the College of Health and Medical Technologies in Sulaimaniyah, Iraq. The isolates were cultured on selective media for identification according to standard protocols (20, 21), and the encoded genes of the antimicrobial resistance and integrons were analyzed using PCR.

### DNA extraction

DNA extraction from the gram-negative bacteria was performed by a boiling method described by Dashti et al. (22). Briefly, a colony of the isolates grown on the selective media was dissolved in 150 µL of sterilized water in a test tube. The test tube was then placed in a water bath for 15 min. After boiling, the test tube content was centrifuged at 10,000 rpm for 5 min. The supernatant was then separated, and 5 µL was used for the molecular analysis.

### PCR analysis of the antimicrobial genes and the Integron-integrases

The investigation into the existence and prevalence of integrons and antimicrobial genes was performed using multiplex PCR for the integrase genes and each of the resistance enzyme groups, as shown in Tables 1 and 2.

**TABLE 1:** DNA sequences of primer combination for the integrases of class 1, class 2, and class 3 integrons

Integrons	Primer ID	Primer sequence (5′---3′)	Amplicon	Reference
Integrase genes	*intI1-*F	CTGCGTTCGGTCAAGGTTCT	882	(23)
	*intI1*-R	GGAATGGCCGAGCAGATCCT		
	*intI2*-F	CACGGATATGCGACAAAAAGGT	746	(24)
	*intI2*-R	GTAGCAAACGAGTGACGAAATG		
	*intI3*-F	GCCTCCGGCAGCGACTTTCAG	939	(24)
	*intI3*-R	ACGGATCTGCCAAACCTGACT		

**TABLE 2:** DNA sequences of primers of ARGs of commonly used antimicrobial drugs investigated in this study

Enzyme	Primer ID	Primer sequence (5′---3′)	Amplicon	Reference
Beta-lactamase resistance genes	*blaTEM-F*	ATAAAATTCTTGAAGACGAAA	1080	(25)
	*blaTEM-R*	GACAGTTACCAATGCTTAATC		
	*blaCMY-F*	GACAGCCTCTTTCTCCACA	1000	(26)
	*blaCMY-R*	TGGAACGAAGGCTACGTA		
	*blaShv-F*	TTATCTCCCTGTTAGCCACC	800	(27)
	*blaShv-R*	GATTTGCTGATTTCGCTCGG		
	*blaOxa-F*	TCAACTTTCAAGATCGCA	610	(28)
	*blaOxa-R*	GTGTGTTTAGAATGGTGA		
	*blaCTX-F*	CGCTTTGCGATGTGCAG	550	(28)
	*blaCTX-R*	ACCGCGATATCGTTGGT		
Tetracycline resistance genes	*tetA-F*	GTAATTCTGAGCACTGTCGC	937	(29)
	*tetA-R*	CTGCCTGGACAACATTGCTT		
	*tetB-F*	CTCAGTATTCCAAGCCTTTG	416	(30)
	*tetB-R*	ACTCCCCTGAGCTTGAGGGG		
	*tetK-F*	TTAGGTGAAGGGTTAGGTCC	718	(31)
	*tetK-R*	GCAAACTCATTCCAGAAGCA		
	*tetM-F*	GTTAAATAGTGTTCTTGGAG	657	(31)
	*tetM-R*	CTAAGATATGGCTCTAACAA		
Quinolone resistance gene	*qnrB-F*	GATCGTGAAAGCCAGAAAGG	469	(32)
	*qnrB-R*	ACGATGCCTGGTAGTTGTCC		
Aminoglycoside resistance genes	*aac6-F*	TGACCAACAGCAACGATTCC	554	(33, 34)
	*aac6-R*	TTAGGCATCACTGCGTGTTC		
	*aac1-F*	ATGGGCATCATTCGCACATGTAGG	873	(35)
	*aac1-R*	TTAGGTGGCGGTACTTGGGTC		
	*aac2-F*	ATGCATACGCGGAAGGCAATAAC	861	(35)
	*aac2-R*	CTAACCGGAAGGCTCGCAAG		
Sulfonamide resistance gene	*sulI-F*	GTGACGGTGTTCGGCATTCT	779	(36)
	*sulI-R*	TCCGAGAAGGTGATTGCGCT		

The reaction mixture of the multiplex PCR contained 10 µL RedTaq DNA polymerase 2× premix, 0.25 µM forward and reverse primers of the integrase genes, 2 µL of DNA extract from each isolate, with the total volume made up to 20 µL with sterilized water. The following conditions were used for the PCR: 94°C for 5 min, followed by 35 cycles of 94°C for 30 s, 55°C for 30 s, and 72°C for 30 s, with a final extension at 72°C for 7 min. Then the PCR was analyzed with a 1% agarose gel using DNA gel electrophoresis at 100 V for 30 minutes. The gel was finally visualized under blue light using SmartDoc 2.0 Imaging System (Accuris, NJ, USA). The multiplex PCR mixture for the antimicrobial genes follows the same combination but with primers from the resistance enzyme group.

Multiplex PCR was performed to amplify multiple β-lactamase resistance genes in a total reaction volume of 20  µL. Each reaction mixture contained 10 µL of 2× RedTaq DNA Polymerase Premix (SBSbio, Beijing, China), 0.25  µM of each primer set, and 2  µL of template DNA. The PCR cycling conditions were as follows: an initial denaturation at 95°C for 5 min, followed by 35 cycles of denaturation at 95°C for 30 s, annealing at 55°C for 30 s, and extension at 72°C for 30 s. A final extension was carried out at 72°C for 7 min.

PCR products were separated by electrophoresis on a 1% agarose gel at 100 V for 30 min. DNA bands were visualized under blue light using the SmartDoc 2.0 Imaging System (Accuris Instruments, NJ, USA).

Five common ESBL genes were targeted: *bla*TEM-1 (1,080 bp), *bla*CMY (1,000 bp), *bla*SHV (800 bp), *bla*OXA (610 bp), and *bla*CTX (550 bp). The genes were divided into two groups for amplification in separate multiplex PCRs: group 1 included *bla*TEM-1 and *bla*CMY, and group 2 included *bla*SHV, *bla*OXA, and *bla*CTX.

### DNA sequencing of the amplicons of *IntI*2

End-point PCR was employed to generate amplicons of the *intI2* gene from isolates that tested positive in the multiplex PCR described above. The reaction mixture was prepared similarly to the multiplex PCR but with specific primers targeting *intI2*, and all reagent volumes were doubled to achieve a final volume of 50 µL. PCR products were separated on a 1% agarose gel via electrophoresis and subsequently purified using a DNA Gel Extraction Kit (SBS Genentech, Beijing, China).

Purified amplicons were submitted for bidirectional sequencing to Macrogen Inc. (Seoul, South Korea), where both DNA strands were sequenced using the forward and reverse primers. The resulting sequences were analyzed using the Omega3DNA sequence analyzer, and the consensus sequences were compared to existing entries in the NCBI GenBank database using the BLAST algorithm to confirm identity and similarity to known integron integrase 2 (*intI2*) gene sequences globally.

## RESULTS

This study was implemented to investigate the prevalence of the three clinically important classes of integrons and characterize them. In addition, a total of 14 antimicrobial genes that could be part of the cassette array, the variable region, of the integrons being circulated among the environmental bacteria in Sulaymaniyah were molecularly screened and analyzed. The possible cassette array investigated includes: five beta-lactamase genes (*bla*TEM*, bla*CTX-M*, bla*SHV*, bla*OXA, and *bla*CMY); four tetracycline resistance genes (*tetA*, *tetB*, *tetK*, and *tetM*); three aminoglycoside resistance genes (*aac1*, *aac2*, and *aac6*); one quinolone resistance gene (*qnrB*), and one sulfonamide resistance gene (*sul1*).

### The prevalence of integron classes 1, 2, and 3 in the environmental microbiota of Sulaymaniyah

**Fig 1:**
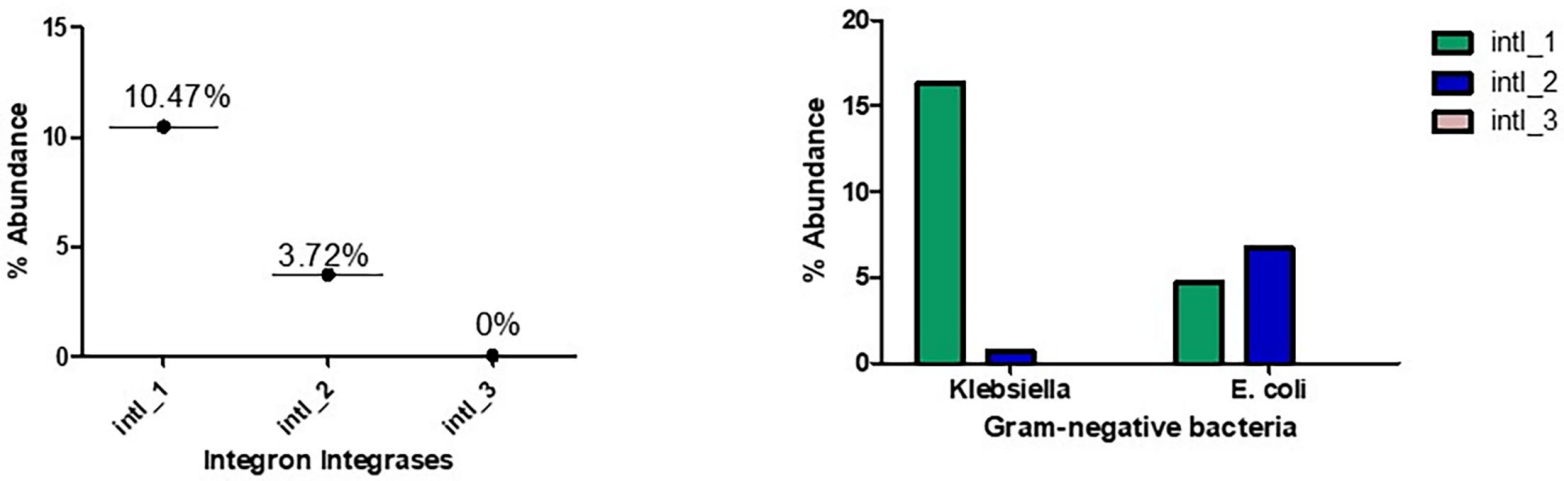
The percentage abundance of integron-integrases in the isolates from Sulaymaniyah and their distribution in the gram-negative bacteria. Integron-integrase 1 is the most abundant in Sulaymaniyah and is mostly in *K. pneumoniae*.

To establish the rate of integron-integrase in *Enterobacteriaceae* in the province, 296 *E. coli* and *K. pneumonia*e isolates from environmental bacteria were investigated. The screening, analyzed by multiplex PCR using integrase gene primers for *intI1*, *intI2*, and *intI3,* revealed the presence of class 1 and class 2 integron-integrases, but no class 3 integron-integrase was detected (Fig. 1). This is not surprising because at the time of writing this manuscript, class 3 integrons were rarely reported in *E. coli and K. pneumoniae* globally (11). In this study, class 1 integron, *intl1*, was the most abundant, with a prevalence rate of 10.47% (31/296), followed by class 2, with a lower rate of 3.72% (11/296) Fig. 1A. In the individual bacterium, it was revealed, as shown in Fig. 1B, that the rate of *intl1* is higher in *K. pneumoniae* at 16.3% (24/147) than in *E. coli* at 4.6% (7/149). Conversely, the rate of *intl2* is higher in *E. coli* with 6.7% (10/149) compared to 0.6% (1/147) obtained in the *K. pneumoniae* isolates (Table 3).

**TABLE 3:** Suspected complete class 1 integrons in *K. pneumoniae* and *E. coli*^,^^*a*^

Bacteria	Sample ID	5′ CS	Variable region	3′ CS	Cassette array
*K. pneumoniae*	K7	*intI1*	*bla*CTX*, bla*OXA*, tetB*	*sul1*	3
	K9	*intI1*	*bla*OXA*, bla*SHV*, tetB*	*sul1*	3
	K69	*intI1*	*bla*CTX*, tetB*	*sul1*	2
	K76	*intI1*	*tetB*	*sul1*	1
	K82	*intI1*	*bla*CTX*, aac2*	*sul1*	2
	K88	*intI1*	Null^*b*^	*sul1*	Null
	K89	*intI1*	*tetA*	*sul1*	1
	K98	*intI1*	*bla*OXA*, bla*TEM	*sul1*	2
	K106	*intI1*	*bla*CTX*, bla*CMY*, tetB*	*sul1*	3
	K133	*intI1*	*bla*CTX*, tetA, aac2, qnrB*	*sul1*	4
	K134	*intI1*	*bla*CTX*, tetB, qnrB*	*sul1*	3
	K140	*intI1*	*bla*CTX*, tetB*	*sul1*	2
	K175	*intI1*	*bla*CTX	*sul1*	1
	K185	*intI1*	*bla*CTX*, tetB*	*sul1*	2
*E. coli*	E11	*intI1*	*bla*CTX*, bla*OXA*, bla*CMY*,*	*sul1*	3
	E71	*intI1*	*bla*CTX*, bla*OXA*, tetB*	*sul1*	3
	E94	*intI1*	*bla*CTX*, bla*OXA*,*	*sul1*	2

^*a*^ Fourteen class 1 integrons were identified in K. *pneumoniae*, making it more potent in MDR than *E. coli* in Sulamaniyah province of Iraq.

^*b*^ Null is the integron integrase that has the 5′ CS and the 3′ CS region but no antimicrobial genes.

### Prevalence of ARGs from the enterobacteria isolates in Sulaymaniyah province

**Fig 2:**
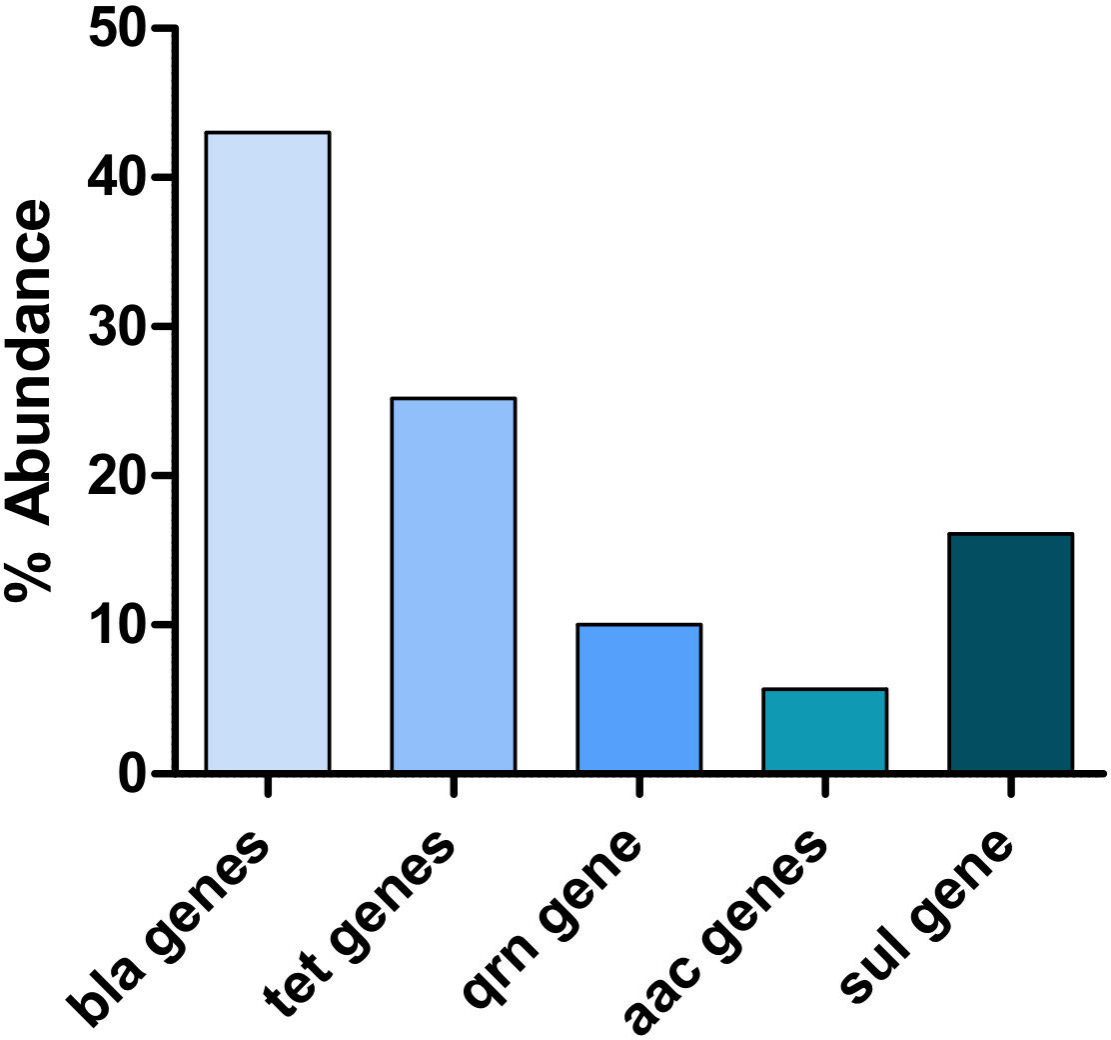
The abundance of antimicrobial genes from the isolates was determined through the multiplex PCR. The five β-lactam resistance genes (bla genes) generated the most ARGs, followed by three of the four tetracycline resistance genes (tet genes). Two of the three aminoglycoside resistance genes (aac genes) generated the least ARGs.

Out of the 296 isolates examined, only 44 (14.86%) did not carry any antimicrobial genes, while the remaining 252 (85.14%) isolates were fortified with multiple ARGs. A total of 663 resistance gene amplicons, 343 from *K. pneumoniae* and 320 from *E. coli*, were successfully detected from the 252 enterobacterial isolates. Figure 2 shows that beta-lactamase resistance genes were the most prominent, with 285/663 (42.99%), followed by tetracycline resistance genes with 167/663 (25.19%) and sulfonamide resistance genes with 107/663 (16.14%). Aminoglycoside was the lowest with just 38/663 (5.73%) detected and followed by the quinolone resistance gene, the resistance gene encoding the enzyme deactivating ciprofloxacin (37), with 66/663 (9.95%).

**Fig 3:**
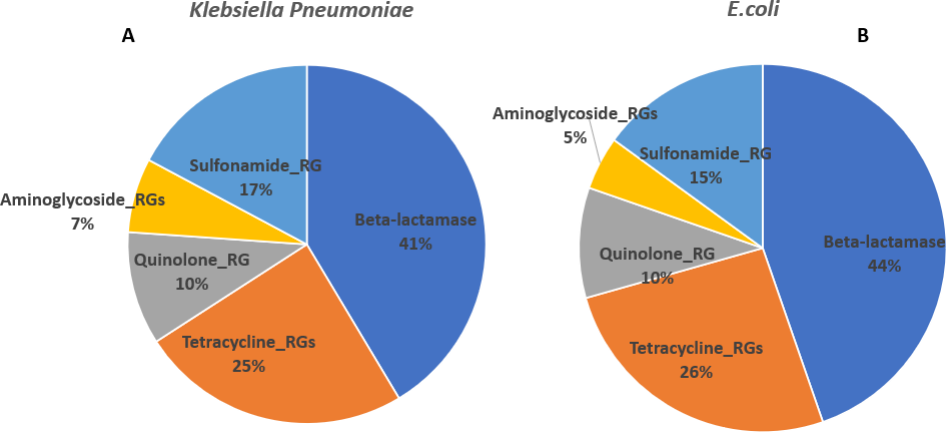
Percentage distributions of ARGs of commonly used antibiotics detected in *K. pneumoniae* and *E. coli* isolates. Panel **A** shows the percentage distributions of the antimicrobial genes in *K. pneumoniae,* very similar to those in *E. coli* (**B**).

The acquisition of antimicrobial genes in both *K. pneumoniae* and *E. coli* was similar, as shown in Fig. 3A and B, indicating the circulation of common drug resistance genes within the environment. All five beta-lactamases constitute 41% and 44% of the ARGs identified in *K. pneumoniae* and *E. coli*, respectively. Tetracycline resistance genes shared 25% and 26% ARG population in *K. pneumoniae* and *E. coli*, respectively, but *tetK* and *tetM* primers did not amplify any amplicon in the isolates. Similarly, only two out of three aminoglycoside genes examined were positive. The *aac6* primers did not amplify. The two genes made up 7% and 5% population in *K. pneumoniae* and *E. coli*, respectively. Sulfonamide resistance genes were 17% and 15% in *K. pneumoniae* and *E. coli*, respectively, and quinolone resistance genes were both 10%.

### Abundance of ESBLs in *K. pneumoniae* and *E. coli* isolates

**Fig 4:**
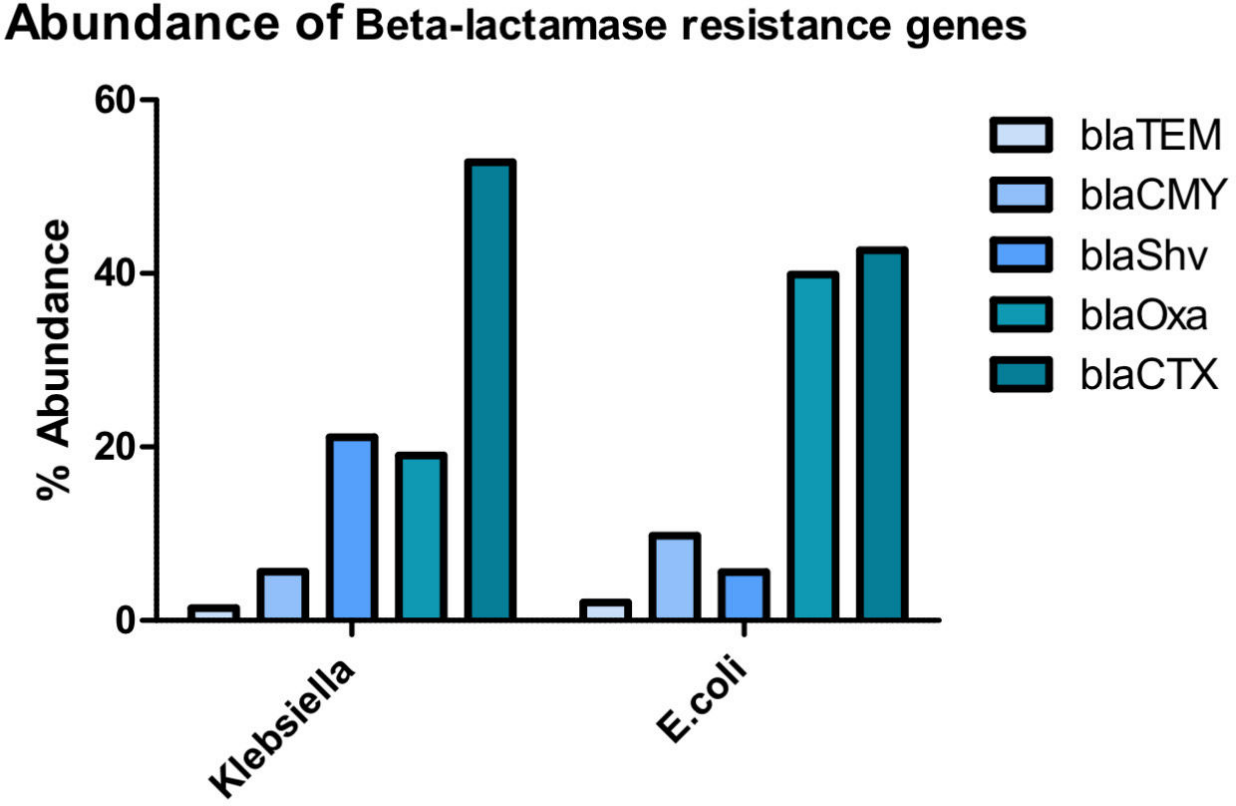
ESBL distribution in the enterobacteria isolated in Sulaymaniyah province of Iraq.

The four ESBL genes of β-lactamases were prominent in the two endobacteria (Fig. 4). *bla*CTX was the most detected, with 75/142 (53%) detected in *K. pneumoniae* and 61/143 (43%) in *E. coli* isolates. *bla*Shv abundance was 30/142 (21%) and 8/143 (6%) in *K. pneumoniae* and *E. coli*, respectively. *bla*CMY was 8/142 (6%) in *K. pneumoniae* and 14/143 (10%) in *E. coli. bla*TEM was the least detected with just 2/142 (1.4%) and 3/143 (2.1%) in *K. pneumoniae* and *E. coli*, respectively. The presence of these ESBL genes poses a high risk for multiple beta-lactam antibiotics, even some of the important drugs like penicillin and cephalosporins (38, 39).

### Multiple resistance genes and stimulation of schematics of a probable complete class 1 integron in the isolates

**Fig 5:**
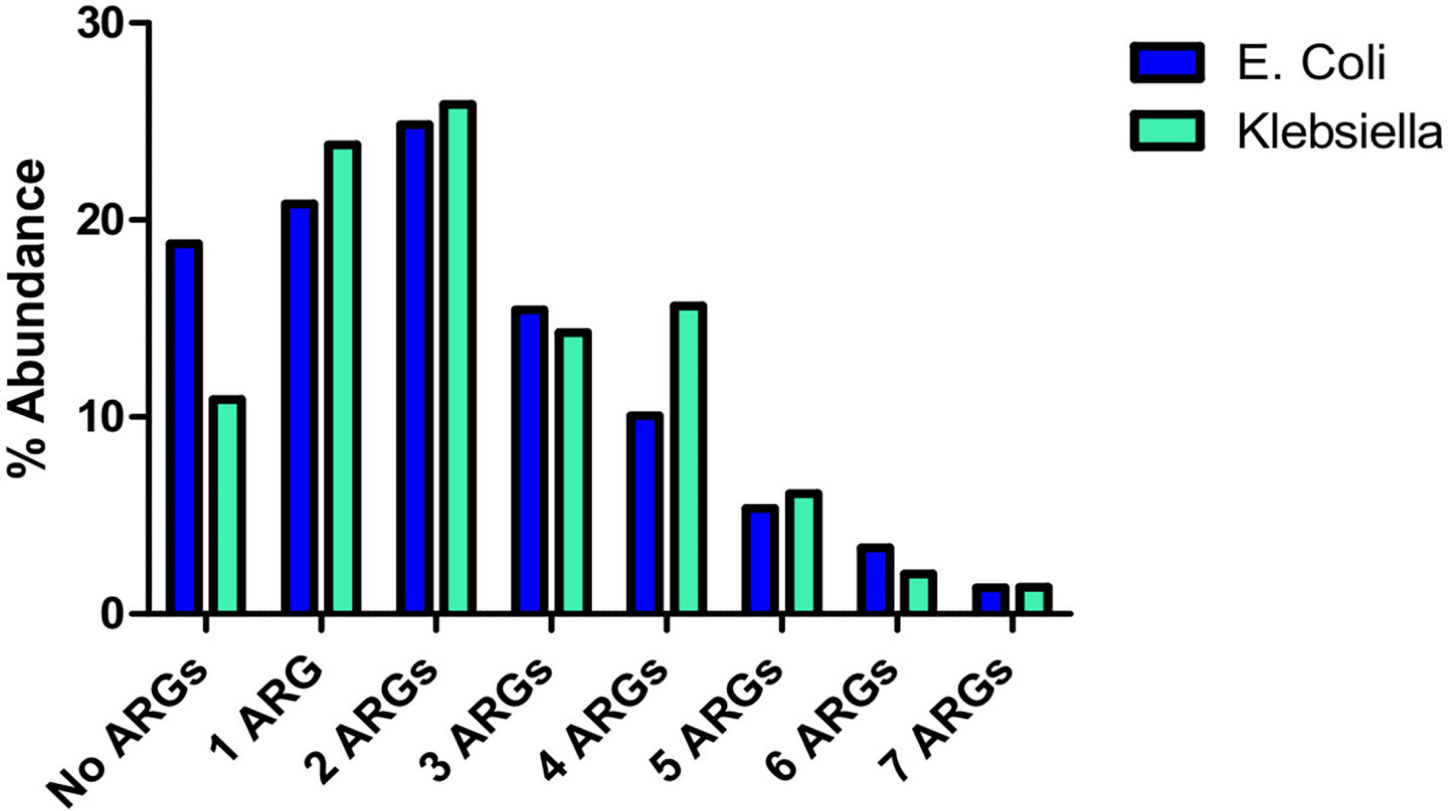
Multiple ARG detection in the isolates of *K. pneumoniae* and *E. coli. K. pneumoniae* isolates have a higher number of multiple ARGS, with just 10.8% susceptible to antimicrobial agents, whose resistance genes were investigated in this study, while 18.79% of *E. coli* were susceptible.

**Fig 6:**
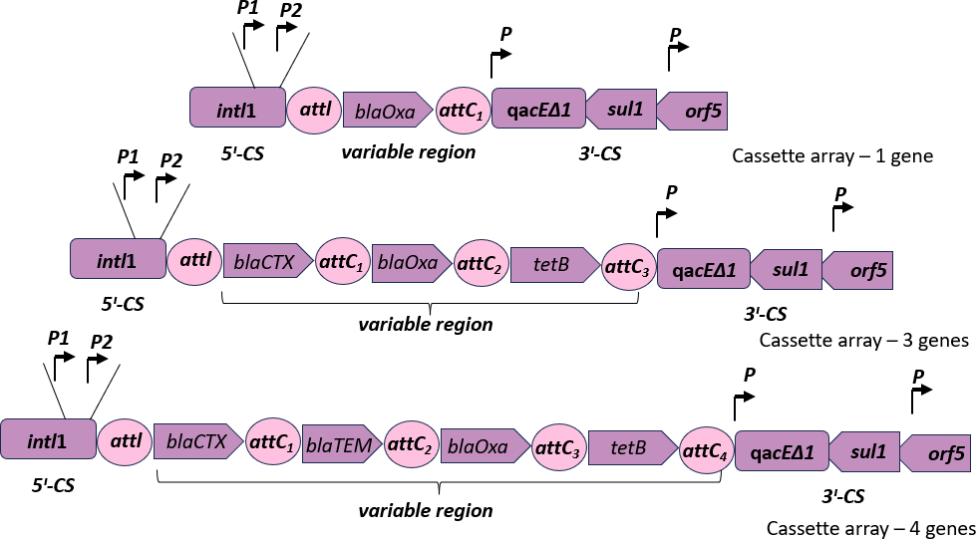
Schematics of probable complete class 1 integrons in *E. coli*. Three class 1 integrons with 1, 3, and 4 cassette arrays were suspected to be in the *E. coli* isolates.

**Fig 7:**
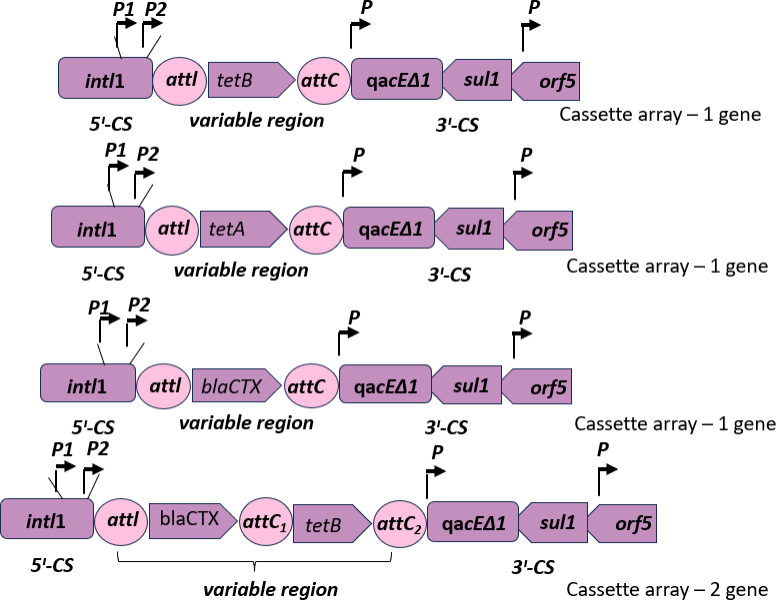
Schematics of probable complete class 1 integrons in *K. pneumoniae*. Twelve probable class 1 integrons were identified, making *K. pneumoniae* more potent in multidrug resistance (MDR) than *E. coli* in Sulamaniyah province of Iraq.

*K. pneumoniae* isolates have more multiple resistance genes than the *E. coli* isolates, as shown in Fig. 5. Some have up to seven different resistance genes, which could constitute a potential gene cassette for integron compositions. Integron class 1 was constituted as per the schematic presented by Deng et al. (11) for the isolates that have the complete components: integron-integrase 1 gene representing the 5′ conserved region and *sul1 gene* for 3′ conserved region. The *qaEΔ1* and *arf5* of the 3′ were not examined in this experiment but were assumed to be present in order to complete the structure of class 1 integron. Figures 6 and 7 show *E. coli* with their representative class 1 integron and *K. pneumoniae* with 12 possible class 1 integrons, respectively (Table 3).

### DNA sequence alignment of *intI2* sequenced against an integron-integrase 2 template

**Fig 8:**
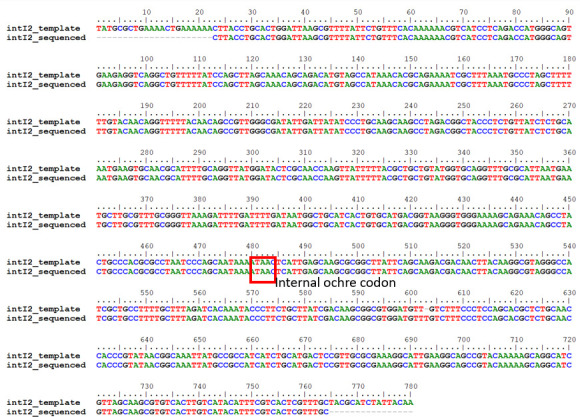
DNA sequence alignment of class 2 integrase from Sulaymaniah with a template from GenBank. The alignment of the template (accession number CP049173) with the samples from this study shows 100% similarity with the signal sequence of *intI2*, the ochre codon boxed in red.

To identify the sequence diversity of *intI2*, the *intI2* gene (five samples) of class 2 integrons was extracted and sequenced. The alignment showed 100% similarity in the DNA of *intI*2 recovered from isolates in Sulaymaniyah with a template already deposited in the database. Most importantly, shown in Fig. 8 is the internal stop codon that makes *intI2* inactive compared to *intI*.

### The phylogenetic tree of class 1, class 2, and class 3 integron integrases

**Fig 9:**
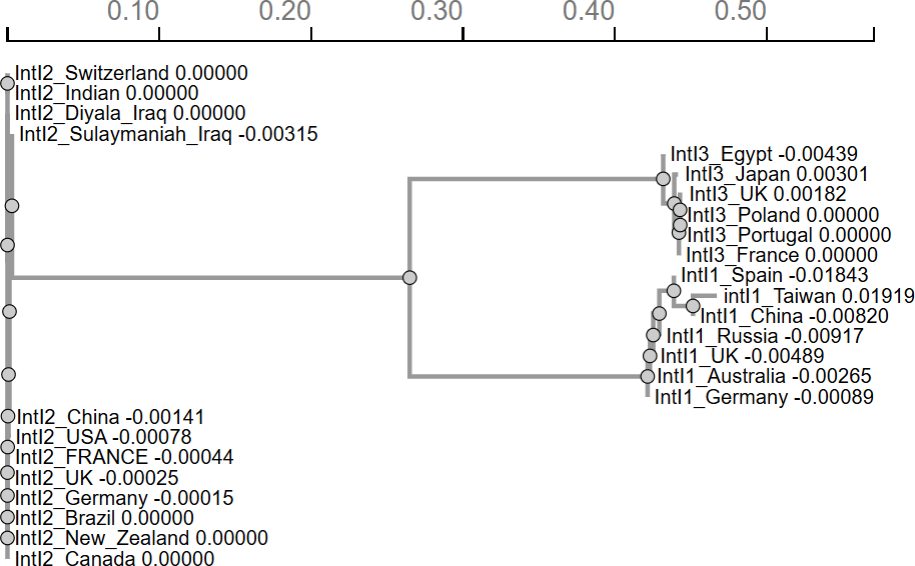
Phylogenetic tree illustrating the evolutionary relationships among the three classes of integron integrases. The *IntI2* sequence is highly conserved across various regions, including Switzerland, India, and Iraq, while the *IntI1* sequence exhibits greater diversity, with notable variations observed in Egypt, Japan, and Australia. The *IntI3* sequence shows moderate conservation, with examples from the United Kingdom and Taiwan. Branch lengths represent genetic distances, with values indicating the degree of divergence.

A phylogenetic tree was constructed for the three classes of the clinical integrons using the EMBL-EBI Job Dispatcher sequence analysis tools (40). Class 2 integrase genes were sequenced, and 11 others from the DNA database were used. Seven class 1 and five class 3 *intI3* were also obtained from the database Table 4. Figure 9 shows that the *intI1* is the most diverse of the integrases examined, followed by class 3 integrase. All 12 class 2 integrases, isolated from different bacteria, including both pathogenic and non-pathogenic bacteria in *Moraxellaceae*, *Pseudomonadaceae*, and *Enterobacteriaceae* members across the globe, have 100% sequence similarity. Hence, this intgra-integrase 2 is highly conserved across the globe.

**TABLE 4:** The accession numbers of the three classes of integron integrases from GenBank for the generation of the phylogenetic tree for the *intI2* sequenced from Sulaymaniah province of Iraq

Integron class	Country	Accession number
Integron-integrase 1	Australia	DQ352176
	Russia	HM569736
	EU722351	EU722351
	Spain	GQ293499
	United Kingdom	CP102212
	Germany	CP151676
	Taiwan	DQ989302
Integron-integrase 2	Sulaymaniah, Iraq	PV929391
	Diyala, Iraq	PP737623
	China	MK670987
	USA	CP141742
	FRANCE	CP102341
	United Kingdom	CP102212
	Indian	CP063843
	Switzerland	CP049173
	Germany	CP151676
	Brazil	CP165845
	New Zealand	CP060709
	Canada	CP128220
	United Kingdom	KX946994
Integron-integrase3	Poland	KF745070
	Egypt	PP730636
	Portugal	AY219651
	France	KT736121
	Japan	AB070224

## DISCUSSION

Antimicrobial resistance in Iraq will continue to increase unabated until the World Health Organization’s Global Antimicrobial Resistance Surveillance System (WHO-GLASS) and regulations on antibiotic stewardship are fully implemented and enforced (18). Continued misuse and improper dispensation of antibiotics are the major driving forces in the development of drug resistance. If no action is taken, it could lead to the defeat of the fight against drug resistance (41). Most provinces in Iraq are now struggling to tackle the overwhelming increase in multiple-drug resistance in clinically important bacteria. In most provinces of Iraq, the susceptibility to the most commonly used antibiotics, including third-generation, is below 20%, and pan-drug resistance is spreading fast (18, 42).

In Sulaimaniyah, the presence of multiple ARGs in more than 80% of isolates, as presented in this study, is worrisome. Previous studies in Iraq have shown that most bacteria are now resistant due to multiple resistance genes acquired to become multidrug-resistant (43, 44). Here, we established some isolates with as many as seven different ARG genes that could render most antibiotics impotent. This will lead to high morbidity and mortality due to difficulties in finding a proper antibiotic for treatment. The presence of both integron classes 1 and 2 up to 20% in the isolates is a dangerous signal that could rapidly spread these ARGs across all the environmental bacteria.

Bacterial class 1 integron can capture and integrate antibiotic resistance genes from different bacterial sources (45–47). Class 1 integron, as a mobile genetic element, can carry and transfer the resistance genes between bacteria via horizontal gene transfer, and this is a leading cause of the increase in resistant bacteria and emergence of new multidrug-resistant bacteria, especially clinical pathogens in hospitals (45–48). Integrons are positioned by nature for better adaptation of bacteria in the environment. Integrons are becoming a major molecular vehicle for the circulation of ARGs in pathogenic bacteria. Their presence may escalate an already bad situation due to drug resistance. It was found in previous studies that more bacteria resistant to antibiotics were associated with class 1 fortified bacteria (49, 50). This is in agreement with the data presented in this study.

According to this study, of the two enterobacteria, *K. pneumoniae* is more dangerous than *E. coli* isolated from the environmental microbiota of Sulaymaniyah province. *K. pneumoniae* causes pneumonia, urinary tract infection, bloodstream infection, intra-abdominal infection, pyogenic liver abscess, and many other hospital-acquired related diseases (51, 52). *K. pneumoniae* has even become more dangerous in Sulaymaniyah because of the high presence of the ARGs detected and the acquisition of the integron-integrase 1, which is the most active integrase. This study, therefore, reemphasized the importance of full implementation of WHO-GLASS and antibiotic stewardship in Iraq (18). *K. pneumoniae* is becoming more and more resistant. The presence of class 1 integrons means it will not be long before all *K. pneumoniae* are fully resistant and could become a de facto pan-drug resistant.

## Data Availability

The confirmed *intI2* gene sequence was submitted to GenBank under the accession number PV929391.
